# Conclusions in Regard to Gastrotomy

**Published:** 1878-04

**Authors:** 


Gastrotomy.—At a recent meeting of the Académie de Médicine (*Le Mouvement Med.*, 1877, No. 50) M. Cazin read a paper upon this subject, which terminated with the following conclusions: 1. Gastrotomy is applicable to cases of internal strangulation by bands or torsions, and in general when occurring suddenly, and in invagination. 2. It is not necessary to know the exact seat of the difficulty before operating. 3. Tardy operation diminishes the chances of success. 4. When the seat of occlusion is unknown, the incision should be in the median line, and sufficiently long; when known, the incision should be made over the seat of the occlusion, and should be relatively small. 5. In order to find the point of stangulation the operator should have in his mind all the possible causes of the difficulty, and should follow the method of Paosi, which simplifies the manœuvre considerably. 6. During the operation, extreme cleanliness, as in ovariotomy, should be observed, and Lister's method, at the same time antiseptic and antiphlogistic, should be employed; 7. Unless some particular indication arises, the patient should be left in perfect quiet, general and local; that is to say, the intestine should not be disturbed by enemata and purgatives.—*Medical Times.*

